# Adipose Tissues from Human and Bat-Derived Cell Lines Support Ebola Virus Infection

**DOI:** 10.3390/v15091827

**Published:** 2023-08-29

**Authors:** Lauren Garnett, Kaylie N. Tran, Zachary Schiffman, Kristina A. Muise, Quinn E. Fletcher, Yvonne A. Dzal, Anders Leung, Alix Albietz, Bryce M. Warner, Bryan D. Griffin, Darwyn Kobasa, Craig K. R. Willis, James E. Strong

**Affiliations:** 1 Special Pathogens Program, National Microbiology Laboratory Branch, Public Health Agency of Canada, Winnipeg, MB R3E 3R2, Canada; 2 Department of Medical Microbiology and Infectious Diseases, Faculty of Health Sciences, University of Manitoba, Winnipeg, MB R3E 0J9, Canada; 3 Department of Biology and Centre for Forest Interdisciplinary Research, University of Winnipeg, Winnipeg, MB R3B 2E9, Canada; 4 Pediatrics & Child Health, College of Medicine, Faculty of Health Sciences, University of Manitoba, Winnipeg, MB R3A 1S1, Canada

**Keywords:** Ebola virus, Filoviruses, reservoir host, adipose tissue, metabolism

## Abstract

Ebola virus is a zoonotic pathogen with a geographic range covering diverse ecosystems that are home to many potential reservoir species. Although researchers have detected Ebola virus RNA and serological evidence of previous infection in different rodents and bats, the infectious virus has not been isolated. The field is missing critical knowledge about where the virus is maintained between outbreaks, either because the virus is rarely encountered, overlooked during sampling, and/or requires specific unknown conditions that regulate viral expression. This study assessed adipose tissue as a previously overlooked tissue capable of supporting Ebola virus infection. Adipose tissue is a dynamic endocrine organ helping to regulate and coordinate homeostasis, energy metabolism, and neuroendocrine and immune functions. Through in vitro infection of human and bat (*Eptesicus fuscus*) brown adipose tissue cultures using wild-type Ebola virus, this study showed high levels of viral replication for 28 days with no qualitative indicators of cytopathic effects. In addition, alterations in adipocyte metabolism following long-term infection were qualitatively observed through an increase in lipid droplet number while decreasing in size, a harbinger of lipolysis or adipocyte browning. The finding that bat and human adipocytes are susceptible to Ebola virus infection has important implications for potential tissue tropisms that have not yet been investigated. Additionally, the findings suggest how the metabolism of this tissue may play a role in pathogenesis, viral transmission, and/or zoonotic spillover events.

## 1. Introduction

A steady increase in Ebola virus (EBOV) outbreaks in endemic regions and an extremely high disease burden make EBOV an ongoing global public health concern [1]. Unfortunately, a significant gap in knowledge in the Ebolavirus field is the unknown identity of the zoonotic reservoir [2]. Although researchers have discovered viral RNA and serological evidence of past EBOV infection in different rodent and bat species through extensive sampling in endemic regions, no naturally infected hosts harbouring the infectious virus have been identified [3,4,5,6,7,8,9,10]. Considering the extensive efforts invested in identifying the reservoir host, it can be concluded that the reservoir is either overlooked during sampling, is rarely encountered, and/or specific or stochastic conditions are needed to regulate re-emergence events. To assist in closing this gap in the EBOV field, we aimed to investigate the hypothesis that adipose tissue is a previously overlooked, unique tissue tropism for EBOV infection, which may affect or be affected by the metabolism of the tissue.

Historically, adipose tissue, occupying 4% to over 40% of a mammal’s body composition, was predominately viewed as an inert reservoir for energy storage [11]. However, this previously underappreciated tissue, comprised of adipocytes and the stromal vascular fraction (SVF-preadipocytes, endothelial cells, lymphatic vessels, nerve tissue, and immune cells), is a complex endocrine organ helping regulate homeostatic systems and whole-body metabolism [12]. Mammals possess three main types of adipose tissue: white adipose tissue (WAT), brown adipose tissue (BAT), and beige adipose tissue (also referred to as brite), each having distinct origins, cell morphology, tissue distribution and physiological function (thoroughly reviewed by Rosenwald, M et al. [13]).

Adipose tissue can harbour pathogens which may benefit from the niche environment, consequently altering host immune cell activation, adipocyte inflammation, and dysfunction, leading to alterations in overall metabolic health [14,15,16,17,18]. However, little is known about the interaction between EBOV and adipose tissue, during either acute or persistent infection, or the potential alterations in the host’s metabolic profiles that may interplay. The first report of Ebolavirus infection and replication in adipose tissue was in rhesus macaques infected with Reston virus, based on significant numbers of viral particles via electron microscopy in adipocytes lining the pharynx [19]. Similarly, in 1999, researchers reported large amounts of EBOV viral RNA in peritoneal adipose sections from infected guinea pigs [20]. 

Recently, a study showed that white adipocytes are permissible to EBOV infection both in vitro and in vivo, resulting in the upregulation of pro-inflammatory profiles, perhaps playing a role in acute infection pathogenesis [21]. Additionally, the plasma lipidome in humans is drastically affected during and after acute EBOV infection, showing that adipose tissue likely plays a role in the host’s response during critical illness [18]. Although virions can target and replicate within white adipocytes during acute EBOV infection, no evidence suggests that EBOV targets brown adipocytes, or implies that adipose may play a reservoir tissue function during persistent infection. 

Due to increasing evidence of prolonged EBOV infection in human survivors, the related recrudescence and reignition of human-to-human transmission has prompted questions as to the role humans play as reservoir species [22,23]. Although rare, recrudescence in even a small subset of survivors impacts public health. Such consequences were made evident by an outbreak in Guinea in 2021 involving 23 confirmed cases and 12 deaths, in which genomic sequence data implied the outbreak was a reintroduction of the same circulating virus from 2013 to 2016 [22]. Unfortunately, similar to the presumed zoonotic reservoir, the parameters that govern prolonged human infection, re-emergence, and transmission are not well understood.

The interplay between EBOV infection and BAT is of particular interest due to the tissue’s high metabolic capabilities. One of the main physiological functions of BAT is in non-shivering thermogenesis (NST) to help regulate body temperature. NST is regulated by the high concentration of uncoupling protein 1 (UCP1) on the inner mitochondrial membrane. UCP1 uncouples the proton motor force to generate substrate oxidation, enhancing cellular heat output. Comparatively, the proton motor force is normally used to generate ATP for energy through oxidative phosphorylation and glucose catabolism [24]. Many bats, even in the tropics, rely on heterothermy and torpor, storing large reserves of BAT to fuel the metabolic heat production required for arousal from torpor. Thus, reliance on BAT and potential temporal fluctuations in BAT metabolism within the hosts suggest the possibility that BAT, and its associated metabolism, could play a vital role in the maintenance of EBOV in the reservoir(s) and in spillover events. This study aimed to assess if in vitro BAT cultures, including those originating in a species relatively closely related to some of those implicated as potential reservoirs (i.e., bats), are susceptible to acute and prolonged EBOV infection. In addition, we aimed to assess some of the effects that viral infection has on the metabolism of these cells, including changes in uncoupling protein 1 (UCP1) expression and lipid organization. 

## 2. Methodology

### 2.1. In Vitro Brown Adipose Tissue Cultures

**Figure 1:**
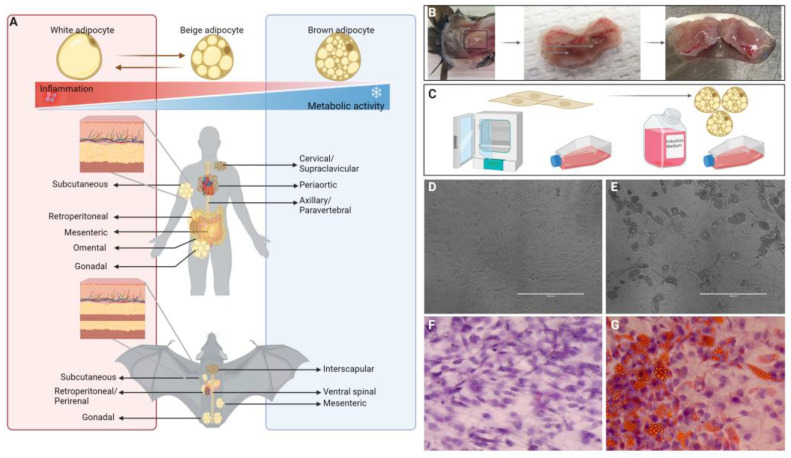
(**A**) Schematic representation of adipose anatomical location in human and bat, including white adipose tissue (left side, outlined in red) and brown adipocyte tissue (right side, outlined in blue) depots. (**B**) Dissection of brown adipose tissue from interscapular region, trimmed to remove as much white adipose tissue and muscle as possible. (**C**) In vitro culturing of primary brown adipocyte methodology. Tissue sections were placed in collagenase and incubated to create single-cell suspensions for culture of pre-adipocytes. Pre-adipocytes were then treated with induction medium (recipe outlined in Section 2) to induce mature brown adipocytes. Microscopy images of primary (**D**) pre-adipocytes and (**E**) induced mature adipocytes at 10× magnification. Oil Red O staining of (**F**) uninduced and (**G**) induced hTERT cells. Figure illustrated in BioRender.

BAT in vitro cultures from two different species were used to evaluate if adipose tissue is susceptible to EBOV infection. Firstly, a commercially available immortalized human BAT cell line, hTERT A41hBAT-SVF (ATCC Manassas, VA, USA CRL-3385), was cultured in Dulbecco’s Modified Eagle’s Medium (DMEM) (Gibco, Waltham, MA, USA 11965-092) supplemented with 10% Fetal Bovine Serum (FBS) (HyClone Waltham, MA, USA SH30396.03), 1% penicillin–streptomycin (Gibco, Waltham, MA, USA 15140-122), and 1% L-glutamine (Gibco, Waltham, MA, USA 25030-081) and incubated at 37 °C and 5% CO_2_. Once cells were confluent, they were induced to mature adipocytes using the protocol outlined below. Secondly, primary cultures of big brown bat (*Eptesicus fuscus*) cells from the interscapular BAT pad (Figure 1) were established. These cells were obtained from female bats originally wild-caught in 2017 within a 200-mile stretch along the North Dakota/Minnesota border. The bats were housed at the University of Winnipeg for two years under their animal Care Protocol AE12193 and weighed between 32.9 and 33.7 g. During dissection of the interscapular BAT pad, tissue was immediately placed into cold isolation buffer following a previously published recipe [25] and transferred on ice to the Public Health Agency of Canada, National Microbiology Laboratory. The tissue was then minced into small sections of approximately 1 mm^3^ and placed into 10 mL of isolation buffer containing 1 mg/mL collagenase II (Gibco, Waltham, MA, USA 17018-029). The tissue was vortexed for 15 s and then placed in a shaking incubator at 37 °C at 150 cycles/minute for 40 min with short vortexing every 10 min. Next, the solution and tissue sections were run through a 100 μm strainer (Thermo Fisher Scientific Waltham, MA, USA 22363549) into a clean 15 mL falcon tube and centrifuged at 300× *g* for 10 min. The supernatant was removed, and the cell pellet was re-suspended in low glucose DMEM supplemented with 10% FBS (HyClone, Waltham, MA, USA SH30396.03), 1% penicillin–streptomycin (Gibco Waltham, MA, USA 15140-122), and 1% L-Glutamine (Gibco, Waltham, MA, USA 25030-081) and plated into a T25 cell culture flask (Corning Inc, Corning, NY, USA 430641U) incubated at 37 °C with 5% CO_2_.

Once pre-adipocyte cultures (human or bat origin) reached 80% confluence, they were split into appropriate plates for further induction and assays. Since mature adipocytes do not replicate, inducing mature adipocytes (Figure 1) was completed once cells reached 100% confluency using a previous published recipe of 0.5 mM (55.57 mg) isobutylmethylxanthine (Millipore Sigma, Burlington, MA, USA I5879), 0.1 μM (19.62 μg) dexamethasone (Millipore Sigma, Burlington, MA, USA D4902), 0.5 μM (1.452 mg) Human insulin (Millipore Sigma, Burlington, MA, USA 1137619001), 2 nM (3.258 μg) 3,3′,5-Triiodo-L-thyronine (T3) (Millipore Sigma, Burlington, MA, USA T-074), 30 μM (5.366 mg) indomethacin (Millipore Sigma, Burlington, MA, USA I7378), 17 μM (1.86 mg) pantothenate (Millipore Sigma, Burlington, MA, USA C8731), 33 μM (4.03 mg) biotin (Millipore Sigma, Burlington, MA, USA B4501) per 500 mL bottle of low glucose DMEM supplemented with 2% FBS (Cytivia Marlborough, MA, USA SH30396.03), and 1% penicillin–streptomycin (Gibco Waltham, MA, USA 15140-122) [25]. Media were changed every 3 days.

### 2.2. Oil Red O

An Oil Red O lipid stain kit (Abcam, Cambridge, UK, ab150678) was used to visualize adipocytes and monitor effective adipocyte induction by staining the triacylglycerol (TAG) droplets and nuclei. Monolayers were washed with PBS before staining. To stain, propylene glycol was added to each well for 2 min, followed by adding the Oil Red O solution for 6 min and then an 85% propylene glycol solution for 1 min. The cells were then washed in distilled water, and hematoxylin stain was added for an additional 2 min, followed by two more washes in distilled water before observation under a microscope.

### 2.3. Uncoupling Protein 1 SYBR Green PCR

Induced and uninduced hTERT A41hBAT-SVF cells were infected with a 0.1 multiplicity of infection (MOI) of wild-type EBOV-GFP Mayinga variant. Cell monolayers were collected at 3, 7, 10, and 14 dpi to assess for any changes in uncoupling protein 1 (UCP1) levels. Briefly, total RNA was extracted from immortalized human adipocytes using the TRIzol lysing reagent and Direct-zol RNA miniprep plus kit (Zymo Research, Irvine, CA, USA R2072) according to manufactures instructions. Total. RNA (50 ng) was reverse transcribed using an oligo(dT)18 primer and Maxima H Minus Reverse Transcriptase kit (Thermo Fisher Scientific, Waltham, MA, USA K1651). PCR was subsequently performed on a thermal cycler (QuantStudio 5, Applied Biosystems, Waltham, MA, USA) using QuantiTect SYBR Green PCR kits (QIAGEN, Hilden, Germany 204154) with previously published primers; hUCP1F: CCGGCGGTCGGTTCA, hUCP1R: CAGGCCCCCCATCTTCA. UCP1 Expression levels were calculated relative to Ribonuclease P expression and reported as fold changes from control samples.

### 2.4. In Vitro Ebola Virus Infection

All in vitro adipose tissue (hTERT and big brown bat BAT) infections were completed using a 0.1 MOI of wild-type EBOV Mayinga with a GFP reporter (EBOV-GFP). All work with live EBOV was completed in a containment level 4 laboratory (CL4). To assess the different adipose tissue cultures’ susceptibility to EBOV infection, viral growth kinetics were completed through quantitative real-time polymerase chain reaction (qRT-PCR) for viral RNA, infectious virus, and green fluorescent protein (GFP) expression.

For assessment of viral RNA levels, supernatant was removed from CL4 via the AVL inactivation protocol. Briefly, 140 µL of the sample was inactivated in 560 µL Buffer AVL for 10 min; then, the contents were transferred to a tube containing 560 µL of 100% ethanol for an additional 10 min. Viral RNA was extracted using the QIAmp viral RNA minikit (QIAGEN, 52906, Hilden, Germany) following the manufacturer’s instructions. 

Viral RNA loads were measured by RT-PCR targeting the EBOV L gene (EBOV_LF: 5′-CAGCCAGCAATTTCTTCCAT-3′ EBOV_LR: 5′TTTCGGTTGCTGTTTCTGTG-3′ EBOV_LP: 5′56-FAM/ATCATTGGC/ZEN/GTACTGGAGCAG/3IABkFQ/3′) using either the Light Cycler 480 RNA Master Hydrolysis Probes kit (Roche, 04991885001 Mannheim, Germany) or TaqPath master mix (Applied Biosystems, Waltham, MA, USA A28523) and run on a QuantStudio 5 RT-PCR system to measure the quantification cycle (C_q_). Statistical significance was determined using the Holm–Sidak method with alpha = 0.05.

Infectious virus titrations were performed by a median tissue culture infectious dose (TCID_50_) assay using Vero E6 cells (ATCC Manassas, VA, USA CRL 1586). Briefly, 10-fold dilutions of the samples were incubated on Vero E6 monolayers maintained in DMEM supplemented with 1% FBS, 1% penicillin–streptomycin, and 1% L-glutamine in triplicate and incubated at 37 °C with 5% CO_2_. Following incubation for 14 days, the cytopathic effect (CPE) was measured under a microscope, and TCID_50_/mL was calculated using the Reed and Muench method as previously described [26]. 

Fluorescent microscopy to visualize GFP expression from wild-type EBOV-GFP infected cells was completed on an EVOS FL microscope using a GFP imaging cube (Thermo Fisher Scientific, Waltham, MA, USA). To quantify GFP expression, fluorescence was read with an excitation of 488 and an emission of 516 on the Synergy HT (BioTek, Winooski, VT, USA) plate reader.

## 3. Results

**Figure 2:**
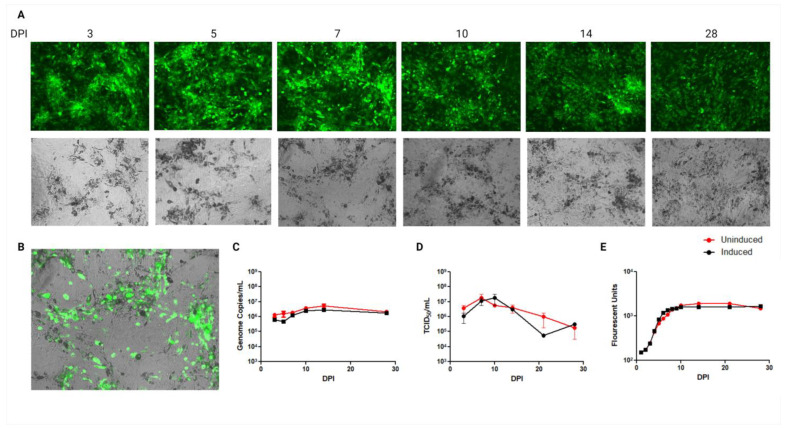
Wild-type EBOV-GFP infection kinetics in human BAT, hTERT A41hBAT-SVF. Induced (black) and uninduced (red) cultures were infected with 0.1MOI wild-type EBOV-GFP Mayinga variant. (**A**) Microscope images at 10× magnification and collection of supernatants for (**C**) RT-PCR and (**D**) TCID_50_ from induced and uninduced hTERT cells were completed on days 3, 5, 7, 10, 14, and 28 dpi; uninduced culture images are not shown. (**B**) An overlayed transmitted and GFP image from induced hTERT infected cells at 7 dpi is shown to indicate better which cells in the culture are infected. (**E**) Plate reads for GFP fluorescent analysis were completed daily until 10 dpi and then again at 14, 21, and 28 dpi.

Initial experiments aimed to evaluate EBOV replication kinetics in adipose tissue in vitro; therefore, a commercially available human BAT cell line, hTERT A41hBAT-SVF, was infected with EBOV-GFP Mayinga variant at an MOI of 0.1. Infection kinetics were evaluated in uninduced (pre-adipocytes) and induced (mature adipocytes) A41hBAT-SVF cultures. Uninduced cultures remained in a fibroblast-like state, whereas induced adipocytes developed lipid TAG droplets that appear as darkened areas under microscopy. There was no substantial difference in viral RNA, infectious virus, or GFP expression between uninduced and induced adipocyte cultures (Figure 2C–E). In all cultures, GFP expression, viral RNA, and infectious titres remained at consistent levels up to 28 dpi, with no visual CPE (Figure 2A). Although both induced and uninduced cultures exhibited similar GFP expression, the highest GFP expression qualitatively overlapped with areas of mature adipocytes, as depicted by the microscope overlay image (Figure 2B).

**Figure 3:**
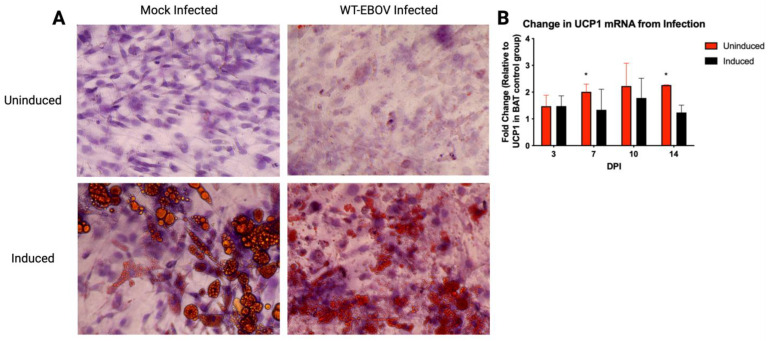
A 40× magnification of hTERT A41hBAT-SVF induced and uninduced cells after either 28days of wild-type EBOV-GFP Mayinga infection or mock infection. (**A**) In mock-infected cells, the uninduced appear as a fibroblast-like cell with no red TAG staining. Alternatively, mock-infected, induced cells show large defined spherical TAG droplets. However, this phenotype is altered by wild-type EBOV (WT-EBOV) infection, in which a limited number of red-stained TAG droplets appeared in the uninduced culture, while the induced culture had reduced TAG droplet size but qualitatively appeared to have more droplets overall, a phenomenon associated with adipocyte browning. (**B**) Changes in UCP1 mRNA over time post-infection for uninduced and induced hTERT A41hBAT-SVF cells expressed relative to uninfected cells. * Significant differences in the uninduced cultures at 7 dpi (*p* = 0.0296) and 14 dpi (*p* = 0.00016).

To assess if infection modified the lipid droplet, an Oil Red O stain was completed on formalin-fixed cultures at 28 dpi. There was a qualitative reduction in size but increase in number of the TAG droplets in the induced infected cultures compared to adipocytes in the induced mock cultures (Figure 3A). The uninduced infected cultures also had qualitatively more lipid TAG droplets compared to the uninduced mock cultures despite not being induced with the differentiation-stimulating culture medium (Figure 3A). Furthermore, a SYBR Green PCR for UCP1 mRNA fold changes was completed on hTERT A41hBAT-SVF cells infected with a 0.1 MOI of wildtype EBOV-GFP collected at 3, 7, 10, and 14 dpi. Although there was no difference between the infected uninduced and induced cultures, both uninduced and induced hTERT cultures had 1.5–2.5-fold increase in UCP1 mRNA compared to uninfected controls showing significant differences in the uninduced cultures at 7 dpi (*p* = 0.0296) and 14 dpi (*p* = 0.00016) (Figure 3B).

**Figure 4:**
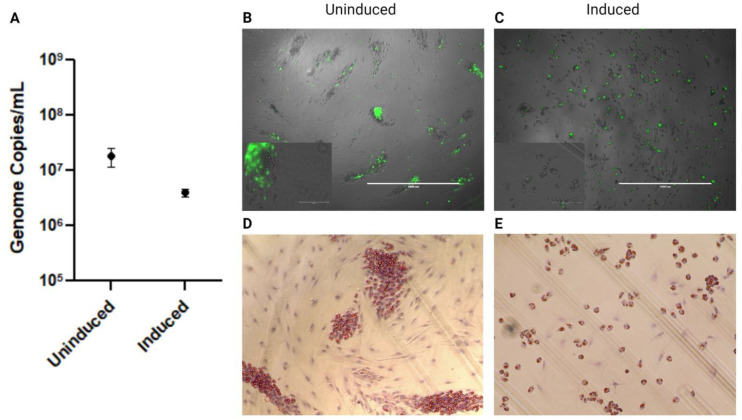
Induced and uninduced primary big brown bat BAT cultures infected with 0.1 MOI wild-type EBOV-GFP Mayinga. Supernatant collected at 3 dpi was assessed for (**A**) viral RNA levels. (**B**,**C**) A 4× magnification overlay between transmitted and GFP channels with an additional 40× magnification showing co-localization of GFP infection and mature adipocytes at 3 dpi. (**D**,**E**) A 20× magnification image of Oil Red O staining of lipid droplets assisting in identifying mature adipocytes to pre-adipocytes.

With the results that human adipose tissue cultures are susceptible to EBOV infection and can persist without causing CPE, it was important to assess if this was also true in cells closely related to the hypothesized reservoir host. Therefore, BAT primary cultures were established from wild-caught big brown bats. Primary big brown bat BAT cells were infected with a 0.1 MOI of wild-type EBOV-GFP Mayinga variant. Supernatant collected at 3 dpi showed high levels of viral RNA in both induced and uninduced cultures (Figure 4A). It should be noted that the induced cultures have reduced cell numbers due to the difficult nature of primary cells under the pressures of induction agents. This difference likely plays a role in the induced cultures having decreased viral RNA levels compared to the uninduced cultures. Unfortunately, infectious titres could not be assessed with the limited yield of cells obtained. GFP expression occurred in both induced and uninduced cultures (Figure 4B,C). However, due to the nature of primary adipose culture, there were areas of induced adipocytes in the uninduced culture, confirmed through Oil Red O staining (Figure 4D). These induced cell areas are associated with high GFP expression (Figure 4B), thereby supporting the claim that induction, whether chemical or spontaneous, conveys EBOV replication.

## 4. Discussion

For this study, we investigated EBOV infection of adipose tissue from human and bat origin to test the hypothesis that fat represents a potential niche tissue reservoir for persistent EBOV infection and to shed light on potential mechanisms underlying interactions between EBOV and adipocytes. Firstly, it aimed to determine if EBOV is capable of infecting adipose tissue, then assess if metabolic alterations occur during infection in vitro. During the initiation of our experiments, in vitro EBOV infection in adipose cells had not been reported. However, since then, a study showed that human white adipocytes are susceptible to acute EBOV infection, which results in a subsequent upregulation in pro-inflammatory cytokine expression [21]. Our results build on these findings by addressing infection kinetics in BAT cultures beyond 120 h revealing the potential of the tissue as a reservoir for prolonged infection. Human adipocytes, specifically brown adipocytes and the associated SVF, were shown to be susceptible to EBOV infection without presenting any obvious cytopathic effects associated with viral replication. The infection retained consistent levels of both viral RNA and infectious virus for 28 days, suggesting that adipose tissue could be a potential target for prolonged EBOV infection even within human populations. It is interesting that there was little difference between the viral release over the course of infection of the induced hTERT cells between days 3 and 28. While the key point here was to show that there was a continuous release of infectious virus throughout this time series with very little associated CPE, it is not known if this expression would have continued beyond 28 days post-infection or how much of the virus readout (Genome copies/mL, TCID_50_/mL, and fluorescence) represents input virus. Future studies will include earlier and later timepoints to answer these queries. While at least some of this may be input virus, it is clear that with the increase from day 3 to day 10 and beyond, as well as the continued release to day 28, this represents replicative virus and continuous release with only a minor level of decrease in infectious titres into day 28 (Figure 2C).

With the finding that EBOV persists within human adipose tissue cultures, an important next step was to assess if this also holds true in adipocytes isolated from animals more closely related to the suspect reservoir species. We used adipocytes from North American big brown bats as a surrogate for those of hypothesized reservoir African fruit or insect-eating species which have been implicated as potential reservoir hosts of EBOV (e.g., Pteropodidae: *Hypsignathus monstrosus*, *Epomops franqueti*, *Myonycteris torquata*; Miniopteridae: *Miniopterus inflatus*; Molossidae: *Mops condylurus*) [5,27]. Although distantly related to potential reservoir hosts, big brown bats are in the same sub-order of at least two potential host species. The finding that bat adipocytes are susceptible to EBOV infection has implications for potential tissue tropisms in reservoir species that have not yet been thoroughly investigated. Although this is a novel finding for EBOV, other zoonotic pathogens have been shown to affect adipose tissues of reservoir hosts. For example, BAT is a potential tissue reservoir for Rabies virus, and there appears to be a direct relationship between metabolic energy expenditure and the pathogenesis of Rabies virus in at least one species [28,29,30]. When pallid bats (*Antrozous pallidus*) were held at 4 °C, a temperature which likely resulted in deep, prolonged torpor and reduced energy expenditure, there were no detectable levels of virus after infection. In comparison, approximately 80% of experimentally infected bats had detectable virus when held at 22 °C and 37 °C, respectively [31]. Similarly, little brown bats (*Myotis lucifugus*) and Mexican free-tailed bats (*Tadarida brasiliensis*) had reduced Rabies viral replication when maintained at temperatures that favour hibernation and torpor (5 °C or 10 °C), with viral replication subsequently increasing when the bats were transferred to warmer temperatures (29 °C) [30]. Another group further confirmed these findings by investigating the effects of torpor in silver-haired bats (*Lasionycteris noctivagans*) with various Rabies virus strains and inoculation routes, finding that bats only developed the disease after being moved out of hibernation into higher temperature environments [32]. Japanese encephalitis virus (JENV) has also been isolated from BAT in both experimentally infected and naturally infected bats of various species [33,34]. Interestingly, as for Rabies, big brown bats experimentally infected with JENV maintained a latent infection for 107 days when housed in simulated hibernation conditions, but viremia occurred three days after bats were moved back to room temperature [35]. From these studies with Rabies and JENV, it is evident that variations in temperature result in changes in viral replication and shedding likely due to downregulated host cellular processes associated with torpid body temperatures for bats and possibly other hibernators. 

More recently, researchers investigating Egyptian fruit bats (*Rousettus aegyptiacus*), the bats that are the suspect reservoir of the related filovirus Marburg virus, showed that experimental infection with Kasokero virus caused disruption of the adipose architecture at the site of infection [36].

We propose an intertwined relationship where fat metabolism is altered by viral infection and/or that the EBOV infection may be affected by changes in fat metabolism. The obvious phenotypic change in the BAT cells from both the human and the bat cultures post-induction and that this phenotypic feature is augmented by infection with EBOV in the uninduced and induced BAT cultures may reflect this interdependence. Treating both the hTERT and big brown BAT cells with induction media caused the lipid droplets to increase in number as well as to become smaller in size. This is a known feature of the transformation from WAT to beige through the browning process. The increase in UCP1 RNA, a precursor event allowing for the dissipation of the proton gradient of the inner mitochondrial membrane and another indication of increased cellular metabolism, that was shown in the hTERT cells over the time course of EBOV infection, supports this claim. Although the approximately 2-fold increase in UCP1 expression may seem relatively small, this change is consistent with upregulated UCP1 levels observed following infections with other pathogens, including Sendai virus, *Mycoplasma* (M.) *pulmonis*, *Heligmosomoides polygyrus*, and Influenza virus [16,37,38]. It is also possible that the decrease in size of the lipid droplets may have resulted from lipolysis following EBOV infection, although this would less likely cause the increase in number of lipid droplets that were seen. Similar metabolic changes have been previously documented during influenza virus and *Trypanosoma cruzi* infection [16,39]. At present we do not know the mechanism by which this increase number of smaller lipid droplets from these cells occurs following EBOV infection, but is an active investigation in our lab. 

Interestingly, the ubiquitous use of fat for energy storage across different animals may result in a generalizable target of infection for a wide range of pathogens and could have implications for understanding both acute and long-term infections as well as for spill-over events. Given that bats are the putative reservoir of EBOV, and that fat, especially BAT, may be a cryptic reservoir tissue within bats, understanding BAT dynamics in potential reservoir species could be crucial for understanding how EBOV remains cryptic but occasionally spills over. For example, little is known about seasonal and/or nocturnal variation in thermoregulation and reliance on BAT in African bats, and a better understanding of this variation could be important. Furthermore, our finding that human fat can also support cryptic infection with EBOV may reveal clues about the apparent re-emergence of EBOV infection from human patients [22,23]. This in vitro study initiated the work into the hypothesis that adipose tissue is a niche tissue reservoir for EBOV infection and could reveal important aspects of the interplay of metabolism and viral kinetics. 

While this study describes EBOV infection parameters in a previously unstudied tissue, there are limitations of this work that should be investigated further. Firstly, the low cell yield collected from big brown bats resulted in small sample sizes restricting any time course analysis of viral growth. Long-term infections in lipid and especially BAT cultures from species more closely related to host species would assist in solidifying the important role adipose tissue plays as a reservoir tissue. Additionally, increased histology staining, including a TUNEL stain for apoptosis would benefit our current qualitative analysis of any cytopathic effects from infection. Finally, there are always limitations to in vitro systems since they fail to show the intricate workings of an in vivo model, including host systemic metabolic functions, inflammatory systems, and confounding counter-regulatory effects. Therefore, future studies will aim to evaluate in vivo modelling of acute and persistent EBOV infection, and the role metabolism plays on disease pathogenesis, outcome, and transmission.

## Data Availability

All data is contained within the article.
